# Microbiota influences host exercise capacity via modulation of skeletal muscle glucose metabolism in mice

**DOI:** 10.1038/s12276-023-01063-4

**Published:** 2023-08-04

**Authors:** Hye Jin Kim, Youn Ju Kim, Yong Jae Kim, Ji Hyeon Baek, Hak Su Kim, Il Yong Kim, Je Kyung Seong

**Affiliations:** 1 Laboratory of Developmental Biology and Genomics, Research Institute for Veterinary Science, College of Veterinary Medicine, Seoul National University, Seoul, 08826 Republic of Korea; 2 Korea Mouse Phenotyping Center (KMPC), Seoul National University, 08826 Seoul, Republic of Korea; 3 BK21 Program for Veterinary Science, College of Veterinary Medicine, Seoul National University, Seoul, 08826 Republic of Korea; 4 Interdisciplinary Program for Bioinformatics, Program for Cancer Biology and BIO-MAX/N-Bio Institute, Seoul National University, Seoul, 08826 Republic of Korea

**Keywords:** Homeostasis, Metabolic syndrome

## Abstract

The microbiota enhances exercise performance and regulates host physiology and energy metabolism by producing beneficial metabolites via bacterial fermentation. In this study, we discovered that germ-free (GF) mice had a reduced capacity for aerobic exercise as well as low oxygen consumption rates and glucose availability. Surprisingly, GF mice showed lower body weight gain and lower fat mass than specific pathogen-free (SPF) mice. Therefore, we hypothesized that these paradoxical phenotypes could be mediated by a compensatory increase in lipolysis in adipose tissues owing to impaired glucose utilization in skeletal muscle. Our data revealed that gut microbiota depletion impairs host aerobic exercise capacity via the deterioration of glucose storage and utilization. The improved browning ability of GF mice may have contributed to the lean phenotype and negatively affected energy generation. These adaptations limit obesity in GF mice but impede their immediate fuel supply during exercise, resulting in decreased exercise performance.

## Gut microbes: unexpected exercise partners

Gut microbes alter metabolism and enhance exercise performance in mice. Gut microbes are known to produce beneficial metabolites and improve health, but how they affect exercise is not well understood. Je Kyung Seong at Seoul National University in South Korea and co-workers compared how mice with a healthy microbiome and germ-free (GF) mice responded to exercise. Although GF mice became exhausted much sooner than non-GF mice, spent one-third less time running, and ate 1.5 times more than non-GF mice, they did not gain weight. Further investigation showed that instead of getting their energy from glucose stored in skeletal muscles, GF mice were burning fat, which reduced their performance; reinstating their gut microbes restored their exercise capacity. A healthy gut microbiome is critical for exercise capacity and future work could focus on identifying key microbial species.

## Introduction

Aerobic capacity is the ability of the cardiorespiratory system to supply sufficient oxygen to peripheral tissues for energy production^1–3^. Therefore, it is regarded as an important fitness factor for maintaining and improving health. Maximal aerobic capacity (VO_2 max_), which serves as an index of cardio-metabolic health, can be increased with aerobic exercise training, such as running, swimming, or cycling^3^. Therefore, methods for maximizing aerobic capacity through exercise training are attracting attention.

Recently, many studies have been performed to understand the interaction between the microbiota and exercise in metabolic health. According to reports, exercise training alters the composition and diversity of the gut microbiota, which in turn leads to metabolic improvement in both humans and rodents^4–9^. Furthermore, exercise enhances the production of microbial metabolites and short-chain fatty acids (SCFAs; such as acetate, butyrate, and propionate)^4,5,10–12^, which play an important role in host energy metabolism through both local and systemic actions on multiple tissues^10,13^. Emerging evidence shows that SCFAs promote skeletal muscle metabolic function and endurance exercise capacity^10,11^. An animal study demonstrated that voluntary exercise increases the n-butyrate concentration in the rat cecum^12^, and intestinal acetate production is an energy source for exercise in the skeletal muscle of mice^5^. A study on humans also showed that exercise training increased fecal concentrations of acetate, propionate, and butyrate in lean but not obese subjects^14^. Transplantation of exercise-conditioned human fecal microbes into mice confers the metabolic benefits of exercise on glucose homeostasis^4^. A well-designed human study showed that microbiota is an essential factor in the development of cardiometabolic diseases^15^. These results suggest that the microbiota is an important mediator and regulator of the benefits of exercise training on metabolic health^4,8,16^. Nevertheless, our knowledge of the mechanisms through which the gut microbiota mediates the effects of exercise and increases host exercise capacity is still limited.

Hsu et al. first reported that endurance swimming time was decreased by half in germ-free (GF) mice compared with that in specific pathogen-free (SPF) mice^17^. It was later confirmed in gnotobiotic models that exercise capacity is conferred by the gut microbiota^18^. However, the authors did not provide convincing physiological and molecular mechanisms to explain the association between gut microbiota depletion and host aerobic exercise capacity. This might be due to the insufficient assessment of systemic metabolic responses in GF mice. Therefore, more investigations are needed to examine the metabolic system of GF mice.

GF mice are known to be resistant to diet-induced obesity and insulin resistance^19–21^. The anti-obesity phenotype of GF mice is associated with enhanced fatty acid metabolism in response to AMP-activated protein kinase (AMPK) activation in the skeletal muscle and liver^19,22^. Glucose deprivation is one factor that activates AMPK, and the activated AMPK cascade increases fatty acid oxidation and glucose uptake in peripheral tissues and reduces gluconeogenesis^23^. This suggests that the increase in fatty acid oxidation by AMPK activation in the peripheral tissues of GF mice may result from glucose deficiency and metabolism.

These results motivated us to investigate the potential of microbiota to modulate glucose metabolism and exercise capacity. We hypothesized that decreased glucose metabolism is associated with impaired exercise capacity in GF mice. To test this hypothesis, we aimed to determine variations in the metabolism and storage capacity of glucose and glycogen, essential substrates for energy supply, in the skeletal muscle of SPF and GF mice. The adaptability of GF mice to voluntary wheel running exercise and the difference in their energy production during an exhaustion test were also determined.

## Materials and methods

### Animals

All mice used in this study were males on the C57BL/6 N background (8 weeks old, 22 ± 2 g) and housed and exercised in a specific pathogen-free barrier facility. GF mice were on the C57BL/6 N background and maintained and exercised in an isolator at the GF facility of Seoul National University, Korea. Both SPF and GF mice were housed at room temperature (23 ± 1 °C) with a relative humidity of 50–60% and a 12 h light/dark cycle with access to a regular chow diet (SPF: NIH-31, GF: NIH-41, Zeigler) and tap water *ad libitum*. The body weights of all mice were measured weekly. Food intake was measured in individually housed mice twice per week. Mice in the exercise groups performed voluntary wheel running for four weeks. The running distance was recorded using a wheel-running activity counter (STARR Life Science).

### Body composition analysis, indirect calorimetry, and exercise respirometry

Body composition (fat mass and lean body mass) was measured using nuclear magnetic resonance (NMR)-based methods (Minispec LF-50, Bruker). For metabolic analyses using indirect calorimetry, the mice were housed individually in cages with free access to food and water under a 12 h light/dark cycle. PhenoMaster 7.5.6 was used to track whole-body O_2_ consumption and CO_2_ generation (TSE Systems). GF mice were monitored using a PhenoMaster Germ-free system (TSE Systems GmbH). The mice used for the exercise respirometry experiment were subjected to the same treadmill protocol (Supplementary Fig. 4a). VO_2_ and VCO_2_ were measured during the exhaustion test using a calo treadmill (TSE system). The test protocol was performed as previously described^24^.

### Metabolic analyses

For the glucose tolerance test, mice that fasted overnight for 16 h were injected intraperitoneally (i.p.) with D-glucose (1 g*kg^−1^ body weight) (G8270, Sigma). For the insulin tolerance test, mice were injected i.p. with 1 U*kg^−1^ body weight of insulin (I-5500, Sigma) after 6 h of fasting. Tail blood was drawn at 15, 30, 60, 90, and 120 min postinjection. Blood glucose was measured using a glucometer (Accu-Check Guide, Roche) at each time point. Serum TG, AST, and ALT levels were measured using a Fuji Dry-Chem NX500i (Fujifilm, Japan). Serum insulin (EZRMI-13k, Millipore), glucose (BM-GLO-100, Biomax), and tissue glycogen (ab65620, Abcam) levels were measured using assay kits. Tail blood lactate was measured with a Lactate Pro 2 (LT-1730, Arkray) at each exercise start and end point. All experiments were performed according to the manufacturer’s instructions.

To measure the energy content in stool, bomb calorimetry was performed using 1 g of dried feces on an isoperibol calorimeter (6400EF, Parr Instrument).

### RNA extraction and quantitative real-time PCR

TRIzol reagent (A33251, Invitrogen) was used to extract total RNA from tissues. A Nanodrop-2000 was used to determine the concentration and quality of RNA (Thermo Fisher). Total RNA (1 g of total RNA) was used to generate cDNA using K-2044-B RT premix and master mix (Bioneer). A QuantStudio5 Real-Time PCR System was used to perform real-time qPCR (Applied Biosystems). A Sensi-Fast SYBR Green Lo-ROX Kit (BIO-94005, Meridian Bioscience) was used to perform PCR in duplicate according to the manufacturer’s instructions. A list of the primer sets used for the mouse target genes is provided in Supplementary Table 1. Primers were purchased from Bioneer. The expression of the target genes was normalized to that of *36b4*. All data are expressed relative to each control value.

### Hematoxylin and eosin staining

Tissues were preserved for 24 h at room temperature in 4% paraformaldehyde (HP2031, Biosesang). Standard techniques were used to slice paraffin-embedded fat sections to obtain 3–4 μm thick tissue specimens, which were subsequently deparaffinized and stained with hematoxylin and eosin. The area of adipocytes was calculated utilizing specific software to determine the cell size. A Pannoramic Scanner (3D HISTECH) and Image-Pro were used to examine all slides (Media Cybernetics).

### Immunohistology

Deparaffinization and hydration of formalin-fixed paraffin-embedded Sections (3–4 μm thick) were followed by antigen retrieval. Anti-UCP1 (ab10983, Abcam) antibody was used to stain beige and brown adipocytes for immunohistochemical analysis. An HRP/DAB detection IHC kit was used to stain all samples for immunohistochemistry (ab64261, Abcam). The Pannoramic Scanner was used to examine all of the slides. All experiments were performed according to the manufacturer’s instructions.

### PAS staining

After fixation, the samples were embedded in paraffin, cut into 3 µm-thick sections, and mounted onto slides. The sections were stained with periodic acid–Schiff (PAS, ab150680, Abcam) for structural assessment of the tissues. All experiments were performed according to the manufacturer’s instructions.

### Western blot analysis

RIPA buffer (BR002, Biosolution) containing protease and phosphatase inhibitors was used to extract total proteins (P3100-001, P3200-001, GenDEPOT). The homogenates were centrifuged for 15 min at 13,000 rpm and 4 °C. A BCA protein assay kit was used to assess the protein concentration in the supernatants (23227, Thermo Scientific). Equal amounts of protein (20–40 μg) were resolved on SDS‒PAGE gels and then transferred to PVDF membranes. The following primary antibodies were used: UCP1 (ab10983, Abcam), OXPHOS cocktail (ab110413, Abcam), α-actin (A2066, Sigma), ATGL (PNPLA2) (#2439, Cell Signaling Technology), p-HSL (ser565) (#4137, CST), HSL (#4107, CST), p-Akt (#9271, CST) and Akt (#9272, CST). The membranes were subsequently treated with a corresponding secondary antibody of either anti-rabbit or anti-mouse IgG (horseradish peroxidase-linked) (PI-1000-1, Vector Laboratories). Enhanced chemiluminescence reagents (170-5061, Bio-Rad, Hercules) were used to visualize the bands, and the signals were evaluated using a Chemi-Doc XRS+ System (Bio-Rad). The concentration of the target protein was then compared to that of β-actin. ImageJ software (NIH) was used to measure band intensities.

### Triglyceride and ATP assays

The levels of skeletal muscle and hepatic triglycerides in the mice were determined using a TG measurement kit (MAK-266, Sigma). All stages were performed in accordance with the manufacturer’s guidelines. ATP levels in the liver and gastrocnemius muscles were evaluated using an ATP Assay Kit (K354-100, Biovision). The level of ATP was measured at 636 nm.

### Statistical analysis

All data are reported as the mean ± standard error of the mean (S.E.M.). Two-tailed Student’s t-tests were used to analyze the differences between two groups. All statistical analyses were performed using SPSS 25 and Prism 7.0. All *p*-values < 0.05 were considered significant. *p < 0.05, **p < 0.01, and ***p < 0.001.

## Results

### Reduced weight gain and improved metabolic parameters in GF mice

**Fig. 1:**
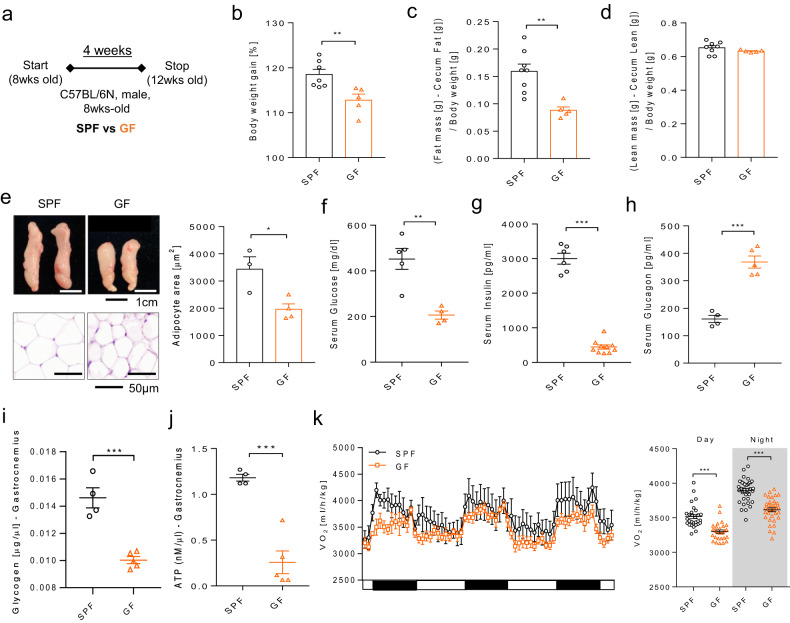
Metabolic phenotype of GF mice compared to SPF mice. **a** Schematic overview of the experiments. **b** Body weight gain (4-week/0-week ratio) [%]; SPF, *n* = 7; GF, *n* = 5. **c** (Fat mass [g] − Cecum fat mass [g]) / Body weight [g]. **d** (Lean mass [g] − Cecum lean mass [g]) / Body weight [g]. **c**, **d** SPF, *n* = 8; GF, *n* = 5. **e** Representative image and H&E staining of gWAT. SPF, *n* = 3; GF, *n* = 4. Scale bar: representative image = 1 cm/H&E = 50 μm. **f** Serum glucose [mg/dl]; SPF, *n* = 5; GF, *n* = 4. **g** Serum insulin [pg/ml]; SPF, *n* = 6; GF, *n* = 10. **h** Serum glucagon [pg/ml]; SPF, *n* = 4; GF, *n* = 5. **i** Glycogen level in gastrocnemius muscle [μg/μl]; SPF, *n* = 4; GF, *n* = 5. **j** ATP level in gastrocnemius muscle [nmol/μl]; SPF, *n* = 4; GF, *n* = 5. **k** VO_2_ level [ml/h/kg]; *n* = 5 for all groups. Values are expressed as the mean ± SEM. Differences between the two groups were analyzed using a two-tailed Student’s *t* test. *p < 0.05, **p < 0.01, ***p < 0.001.

We first investigated the metabolic changes in GF mice for four weeks (Fig. 1a). All measurements were performed under nonfasting conditions. The body weight gain ratio was significantly lower in GF mice than in age-matched SPF mice (Fig. 1b). Supplementary Fig. 1a-c shows that an enlarged cecum and gallbladder comprise the representative phenotype of GF mice^25,26^. Therefore, the body weight and body composition data shown indicate the values after correcting for cecum weight. Body composition results showed that body fat mass was significantly lower in GF mice than in SPF mice (Fig. 1c). The lean mass did not change (Fig. 1d). White adipose tissue (gonadal fat, gWAT) size and adipocyte area were also markedly decreased in GF mice (Fig. 1e). Serum glucose and insulin levels were significantly decreased in GF mice (Fig. 1f, g). Furthermore, glucagon levels were significantly increased in GF mice (Fig. 1h). Serum triglyceride (TG), hepatic TG, and skeletal muscle TG levels were also significantly decreased in GF mice (Supplementary Fig. 1d-f). These results indicated that GF mice had a metabolically healthy phenotype. However, GF mice showed low glycogen and adenosine 5-trisphosphate (ATP) content in the skeletal muscle (Fig. 1i, j). Low glycogen content and ATP levels indicated an energy starved condition in GF mice. As shown in Fig. 1k, the oxygen consumption rate was significantly lower in GF mice than in SPF mice. These results indicated that GF mice had reduced oxidative capacity, which may lead to lower energy availability.

### Physiological response to exercise training in GF and SPF mice

**Fig. 2:**
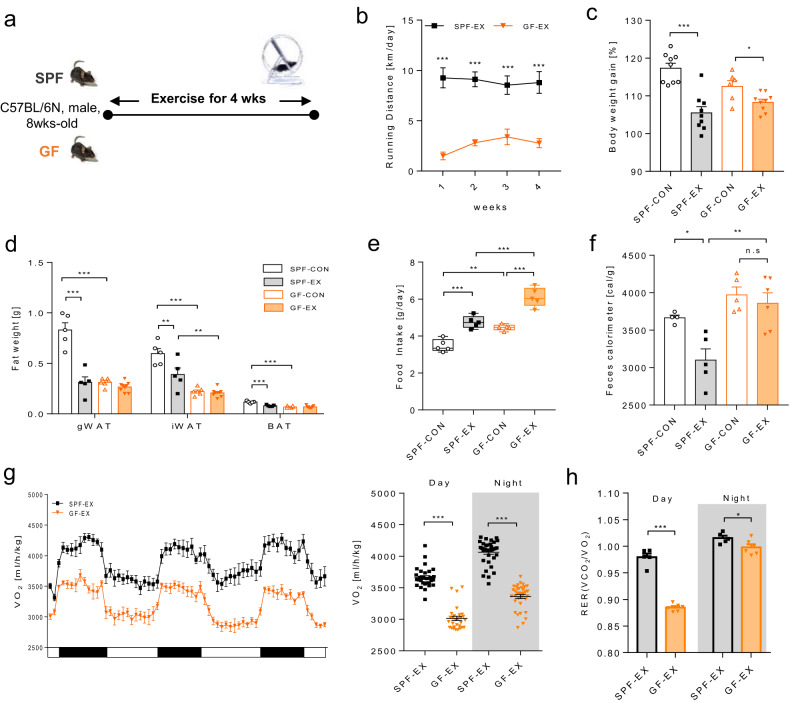
Physiological response to exercise training in GF mice and SPF mice. **a** Schematic overview of the exercise experiments. **b** Daily voluntary wheel running distance [km/day]; SPF-EX, *n* = 5; GF-EX, *n* = 4. **c** Body weight gain (4-week/0-week ratio) [%]; SPF-CON, *n* = 9; SPF-EX, *n* = 9; GF-CON, *n* = 6; GF-EX, *n* = 9. **d** Fat (gWAT, iWAT, BAT) weight [g]; SPF-CON, *n* = 5; SPF-EX, *n* = 5; GF-CON, *n* = 6; GF-EX, *n* = 9. **e** Daily food intake [g/day] during exercise periods; *n* = 5 for all groups. **f** Feces calorimetry after exercise in SPF and GF mice [cal/g]; SPF-CON, *n* = 4; SPF-EX, *n* = 5; GF-CON, *n* = 5; GF-EX, *n* = 6. **g** VO_2_ level [ml/h/kg] after exercise; *n* = 6 for all groups. **h** RER level [VCO_2_/VO_2_] after exercise; SPF-EX, *n* = 5; GF-EX, *n* = 6. Values are expressed as the mean ± SEM. Differences between the groups were analyzed using one-way ANOVA and Tukey’s *post hoc* test. *p < 0.05, **p < 0.01, ***p < 0.001.

To further understand how microbiota depletion modulates host energy availability, we performed voluntary wheel-running exercise training for four weeks in both SPF and GF mice (Fig. 2a). The cecum size did not change as the exercise was performed in a sterile environment (Supplementary Fig. 2a–c). The voluntary wheel-running distance was significantly lower in GF mice than in SPF mice during the training period (Fig. 2b). Although the average exercise amount was reduced by one-third, the weight gain rates of GF-EX mice were similar to those of SPF-EX mice at the end of the intervention (Fig. 2c and Supplementary Fig. 3a–c). The adipose tissue weight of GF-EX mice was also similar to that of SPF-EX mice (Fig. 2d and Supplementary Fig. 3d). Paradoxically, the food intake of GF mice was approximately 1.5 times higher than that of SPF mice during the intervention (Fig. 2e). Body composition results showed that GF mice did not show any exercise-induced changes due to their low basal body fat (Supplementary Fig. 3e–h shows values excluding the cecum weight). As shown in Fig. 2f, the feces of GF-EX mice contained significantly more calories. These results suggest that GF mice may absorb less energy to contribute to exercise due to their higher caloric excretion than SPF mice. This may further explain why GF mice exhibited low body weight gain and adipose tissue weight despite low levels of voluntary exercise and high food intake. Due to these physiological characteristics, GF mice showed a significantly lower VO_2_ rate, RER and activity than SPF mice despite aerobic training (Fig. 2g, h and Supplementary Fig. 3i). We found that GF mice displayed a lower exercise training volume due to restrictions in calorie absorption, which led us to the conclusion that their whole-body aerobic capacity did not improve as much as in SPF mice. Based on these results, we hypothesized that the mechanism of energy utilization could differ between SPF and GF mice.

### GF mice display impaired endurance exercise capacity

**Fig. 3:**
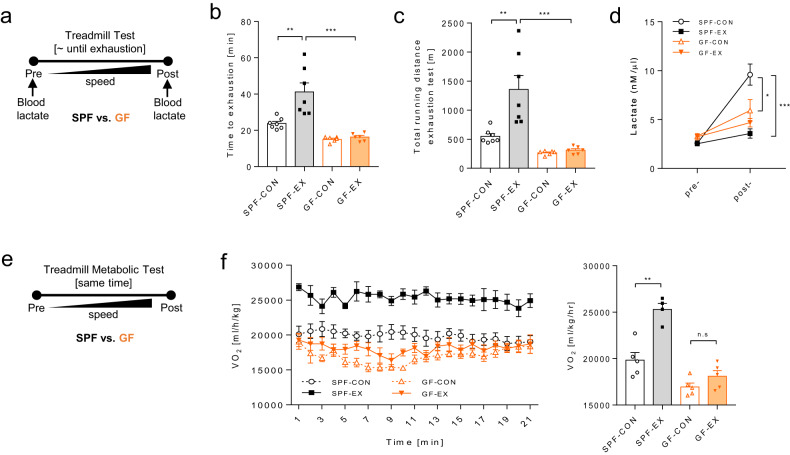
The microbiota controls training-induced changes in exercise capacity and oxygen consumption. **a** Experimental design of the exhaustion test using a treadmill. **b** Time to exhaustion [min]. **c** Total running distance during the exhaustion test [m]. **b**, **c** SPF-CON, *n* = 7; SPF-EX, *n* = 7; GF-CON, *n* = 6; GF-EX, *n* = 6. **d** Serum lactate level [nmol/μl] before and after the exhaustion test; *n* = 6 for all groups. **e** Schematic overview of the exhaustion test using the treadmill metabolic chamber. VO_2_ was measured during exhaustion test periods. **f** VO_2_ [ml/kg/hr] during the exhaustion test. **e**, **f** SPF-CON, *n* = 5; SPF-EX, *n* = 4; GF-CON, *n* = 5; GF-EX, *n* = 5. Values are expressed as the mean ± SEM. Differences between the groups were analyzed using one-way ANOVA and Tukey’s post hoc test. *p < 0.05, **p < 0.01, ***p < 0.001.

A treadmill exhaustion test was conducted to evaluate the difference in exercise performance between SPF and GF mice after voluntary wheel running training had been performed over the four-week period (Fig. 3a and Supplementary Fig. 4). The treadmill exhaustion test results showed that GF mice, with or without exercise training, had a significantly shorter running time and distance until exhaustion than SPF mice (Fig. 3b, c). These results suggested that microbiota deficiency in GF mice impaired improvements in their aerobic exercise capacity. We further measured pretest and posttest blood lactate concentrations to obtain insights into the fuel being utilized for energy production during the exhaustion test. Changes in blood lactate levels before and after the exhaustion test in GF mice showed completely different patterns compared to those in SPF mice (Fig. 3d). We noticed that the blood lactate levels of GF mice did not increase despite running until exhaustion. Many studies have suggested that a low serum lactate concentration after aerobic exercise reflects training adaptation, such as increased oxidative capacity and higher lactate utilization for ATP production^27,28^. Based on these conflicting results, we hypothesized that GF mice have low glucose metabolism capacity.

### Low endurance capacity phenotype of GF mice and defects in respiratory metabolism

To determine the rate of respiratory metabolism during the involuntary treadmill test, mice were subjected to a treadmill exercise test with an indirect calorimetry system (Fig. 3e). During the test, oxygen utilization (VO_2_) significantly increased in trained SPF mice throughout the running period, whereas GF mice showed consistently low VO_2_ during the running period (Fig. 3f). As the exercise-trained GF mice did not show aerobic benefits from the four weeks of wheel-running training due to the short amount of actual exercise time, the lack of exercise capacity in GF mice cannot be easily explained. However, the completely different blood lactate levels and VO_2_ levels between untrained SPF and GF mice in the forced exercise test suggest that glucose metabolism in GF mice entirely differs from that in SPF mice. These results indicate that the microbiota is an essential element in VO_2_ capacity.

### Microbiota depletion impairs host glucose utilization

**Fig. 4:**
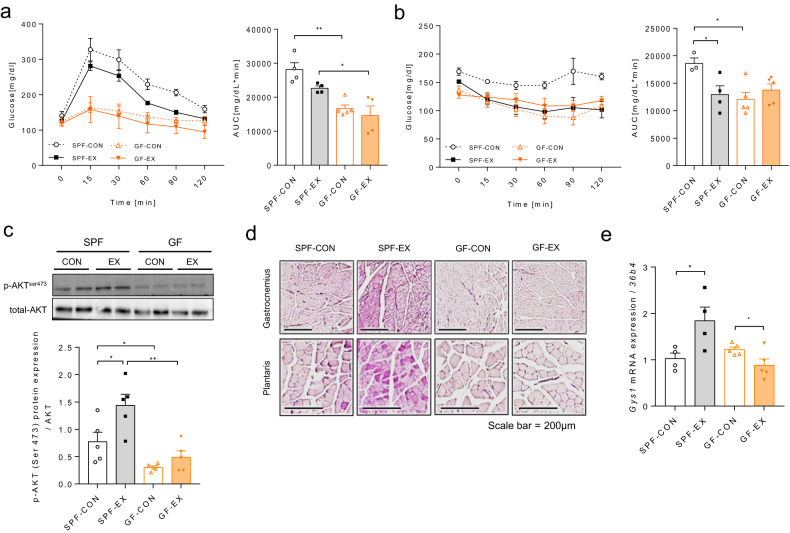
GF mice promote glucose clearance independent of Akt phosphorylation and exhibit abnormal glycogen storage in skeletal muscle. **a** Glucose level during the glucose tolerance test (GTT) [mg/dl] and area under the curve (AUC); SPF-CON, *n* = 4; SPF-EX, *n* = 4; GF-CON, *n* = 5; GF-EX, *n* = 4. **b** Glucose level during the insulin tolerance test (ITT) [mg/dl] and area under the curve (AUC); SPF-CON, *n* = 3; SPF-EX, *n* = 4; GF-CON, *n* = 5; GF-EX, *n* = 5. **c** p-Akt (Ser473) levels of the soleus muscle in exercised SPF and GF mice after insulin (1 U/kg) intraperitoneal (i.p.) injection; *n* = 5 for all groups. **d** PAS staining of gastrocnemius and plantaris muscles; *n* = 3 for all groups; scale bar = 200 μm. **e** *Gys1* gene expression level in gastrocnemius muscle after exercise in SPF and GF mice; SPF-CON, *n* = 4; SPF-EX, *n* = 4; GF-CON, *n* = 5; GF-EX, *n* = 5.

In line with previous work, our data also showed enhanced glucose tolerance and insulin sensitivity in GF mice compared with SPF mice (Fig. 4a, b). A previous study reported that antibiotic-treated (Abx) and GF mice showed improved glucose tolerance and insulin sensitivity due to primary glucose uptake in both inguinal white adipose tissue (iWAT) and gWAT^20^.

Since skeletal muscle is a major site for glucose utilization, studies on metabolism suggest that activation of Akt signaling, specifically in skeletal muscle, leads to systemic glucose tolerance and insulin sensitivity. We examined whether blood glucose clearance in the tissues responsible for it depends on the presence or absence of microbiota in the presence of stimulating insulin. In the skeletal muscle of SPF mice, exercise training increased Akt phosphorylation; interestingly, this effect was completely absent in GF mice (Fig. 4c). However, the phosphorylation of Akt in iWAT was significantly higher in GF mice than in SPF mice (Supplementary Fig. 5). These results suggest that the enhanced glucose tolerance and enhanced insulin sensitivity observed in GF mice were not due to the increased glucose uptake capacity in the muscle typically observed in SPF mice. The browning of iWAT in GF mice can lead to an increase in glucose uptake capacity, according to previous research^20^. We also observed that GF mice displayed iWAT browning (Supplementary Fig. 6). However, there was no direct evidence in our study showing that the browning of iWAT increased glucose uptake, which is a limitation of our study. Nonetheless, our findings imply that the microbiota influenced glucose metabolism in skeletal muscle and adipose tissue by altering the insulin-stimulated Akt signaling pathway.

### Microbiota depletion impairs glycogen storage in skeletal muscle

Skeletal muscle is a major source of energy production during exercise. In particular, glycolysis and the oxidation of muscle glycogen contribute to a higher intensity of exercise^2,29,30^. Therefore, depletion of skeletal muscle glycogen leads to impaired endurance exercise performance. In addition, both aerobic and resistance exercise training significantly increase the activity of insulin-stimulated glycogen synthase (GS).

Based on these facts, we hypothesized that the low oxygen uptake and low Akt activity in the muscle impair glucose absorption and glycolysis capacity, resulting in lower muscle glycogen content in GF mice, which in turn leads to lower aerobic exercise capacity. Periodic acid-Schiff (PAS) staining results showed that four weeks of aerobic exercise training markedly increased the muscle (gastrocnemius and plantaris) glycogen content in SPF mice, whereas GF mice displayed low levels of glycogen (Fig. 4d). Despite exercise training, glycogen synthase 1 (*Gys1*) mRNA levels were downregulated in GF mice (Fig. 4e). Furthermore, exercise training had no effect on the levels of glycolysis-, glycogenesis-, or glycogenolysis-related genes in GF mice compared to SPF mice (Supplementary Fig. 7a–c). These data indicate that depletion of the microbiota lowers glycogen storage capacity and interferes with exercise-induced improvements in glucose absorption and utilization. Additionally, the expression levels of gluconeogenesis-related genes were not increased by exercise training in GF mice (Supplementary Fig. 7d). This indicates that the microbiota regulates the improvement in glucose metabolism that occurs as a result of exercise training and contributes to the improvement in aerobic exercise performance.

### GF mice with SPF microbiota display rescued aerobic endurance exercise capacity as a result of restored glucose metabolism

**Fig. 5:**
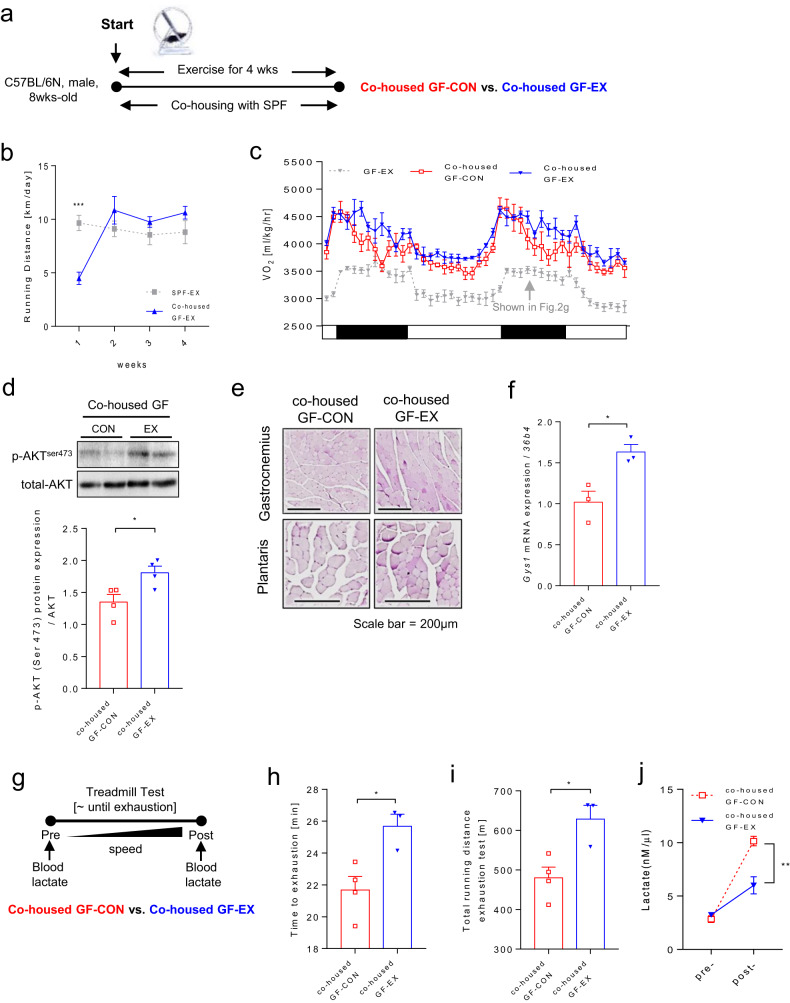
Cohousing GF mice with SPF mice restores glucose metabolism and rescues exercise performance. **a** Schematic representation of the cohousing experiments. **b** Daily voluntary wheel running distance [km/day]; cohoused GF-EX, *n* = 4; SPF-EX (shows repeated values from Fig. 2b). **c** VO_2_ [mL/kg/h] level after exercise under cohousing conditions; *n* = 4 for all groups; GF-EX (show repeated values in Fig. 2g). **d** p-Akt (Ser473) levels of the soleus muscle in exercised cohoused mice; *n* = 4 for all groups. Protein expression was quantified based on Akt protein levels. **e** PAS staining of gastrocnemius and plantaris muscles after exercise in cohoused GF mice; *n* = 3 for all groups. **f** *Gys1* gene expression level in gastrocnemius muscle after exercise in cohoused GF mice; *n* = 3 for all groups. The expression of the target genes was normalized to that of *36b4*. **g** Exhaustion test in cohoused mice after exercise training for 4 weeks. **h** Time to exhaustion [min]. **i** Total running distance during the exhaustion test [m]. **j** Serum lactate level [nM/μL] before and after the exhaustion test. (**g**–**j**) GF-CON cohoused, *n* = 4; GF-EX cohoused, *n* = 3. Values are expressed as the mean ± SEM. Differences between the two groups were analyzed using two-tailed *t* tests. Differences between the groups were analyzed using one-way ANOVA and Tukey’s *post hoc* test. *p < 0.05, **p < 0.01, ***p < 0.001.

To investigate the reversibility of glucose metabolism and exercise capacity, GF mice were cohoused with SPF mice for four weeks (Fig. 5a and Supplementary Fig. 8). The cohoused mice displayed a significantly decreased cecum size (Supplementary Fig. 8a–c) and an increased voluntary wheel running distance compared with GF mice during the four-week training period (Fig. 5b). Furthermore, exercise-trained cohoused mice showed a significantly higher VO_2_ rate, p-Akt level, glycogen content and *Gys1* mRNA level than the untrained cohoused mice (Fig. 5c–f). The rescue experiment clearly showed that the microbiota modulated glucose metabolism in the skeletal muscle (Supplementary Fig. 9). Next, we conducted a forced involuntary treadmill exhaustion test (Fig. 5g). The trained cohoused GF mice had a significantly longer running time (Fig. 5h) and distance (Fig. 5i) until exhaustion than sedentary mice. Changes in blood lactate levels before and after the exhaustion test in cohoused mice followed a pattern that was consistent with that in SPF mice (Fig. 5j). Thus, these results indicated that cohoused mice had restored glucose metabolism. Furthermore, these results emphasize that the microbiota has a significant impact on exercise performance.

### Low glucose metabolism as a result of insufficient training is a characteristic of GF mice

**Fig. 6:**
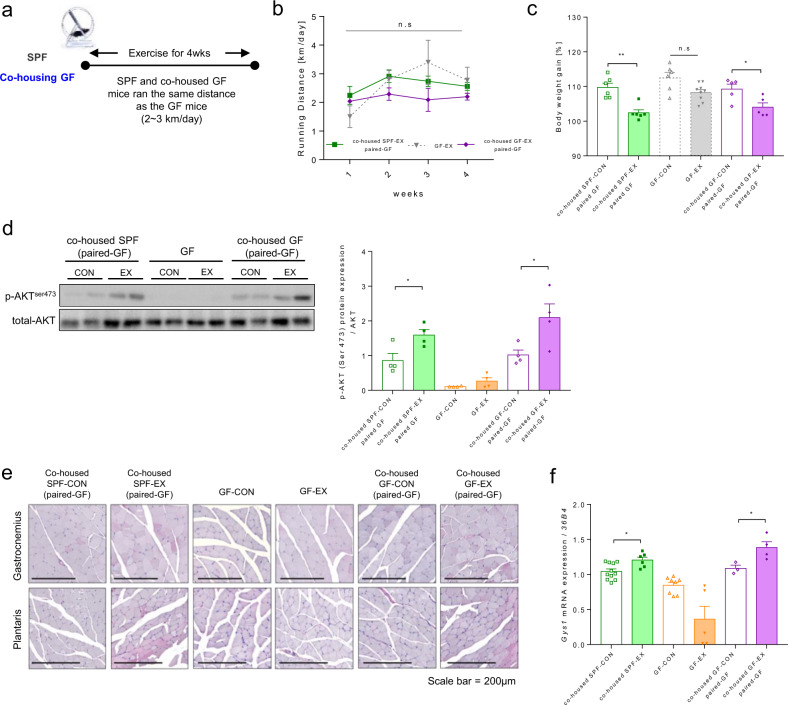
The low glucose metabolism of GF mice is independent of training volume. **a** Experimental design of the exercises performed by SPF and GF mice. **b** Daily voluntary wheel running distance [km/day]; cohoused SPF-EX (paired-GF), *n* = 4; cohoused GF-EX (paired-GF), *n* = 5 (GF-EX, *n* = 4; show repeated values in Fig. 2b). **c** Body weight gain (4-week/0-week ratio) [%]; cohoused SPF-CON (paired-GF), *n* = 6; cohoused SPF-EX (paired-GF), *n* = 6; cohoused GF-CON (paired-GF), *n* = 5; cohoused GF-EX (paired-GF), *n* = 5 (GF-CON, *n* = 6; GF-EX, *n* = 9; show repeated values in Fig. 2c). **d** p-Akt (Ser473) levels in the soleus muscle in cohoused SPF-EX (paired GF), GF, and cohoused GF-EX (paired GF) mice after insulin (1 U/kg) intraperitoneal (i.p.) injection; *n* = 4 for all groups. Protein expression was quantified based on the Akt protein level. **e** PAS staining of gastrocnemius and plantaris muscles after exercise in cohoused GF-EX (paired GF); *n* = 3 for all groups. **f** *Gys1* gene expression level in gastrocnemius muscle after exercise in cohoused GF (paired GF) mice; cohoused SPF-CON (paired GF), *n* = 11; cohoused SPF-EX (paired GF), *n* = 6; GF-CON, *n* = 8; GF-EX, *n* = 5; cohoused GF-CON (paired GF), *n* = 3; cohoused GF-EX (paired GF), *n* = 4. The expression of the target genes was normalized to that of *36b4*. Values are expressed as the mean ± SEM. Differences between two groups were analyzed using two-tailed Student’s *t-*tests. Differences between the groups were analyzed using one-way ANOVA and Tukey’s *post hoc* test. *p < 0.05, **p < 0.01, ***p < 0.001.

Finally, we examined whether the low amount of voluntary aerobic exercise training in GF mice hampered their exercise capacity. As shown in Fig. 6a and Supplementary Fig. 11, we altered the training quantity of SPF mice and cohoused mice based on the voluntary wheel-running distance of GF mice. Despite the short distances ran (Fig. 6b), exercise reduced weight gain in both SPF and cohoused mice (Fig. 6c). Interestingly when SPF and cohoused mice ran the same distance as GF mice, Akt phosphorylation was greatly increased in both groups (Fig. 6d). The results of PAS staining revealed that the lower glycogen storage capacity in the skeletal muscle of GF mice was attributable to the absence of microbiota rather than a lack of training volume (Fig. 6e). The *Gys1* gene expression results also firmly supported this finding (Fig. 6f).

### Microbiota depletion promotes oxidative capacity in adipose tissue

Although one study showed that GF mice have a reduced capacity to resist obesity^31^, other studies showed that microbiota depletion leads to the browning of iWAT, a smaller adipocyte size, and protection against diet-induced obesity^20,21,32,33^.

As shown in Supplementary Fig. 10a, adipose triglyceride lipase (PNPLA2) and hormone sensitive lipase (HSL) phosphorylation levels were significantly increased in both the iWAT and gWAT of GF mice. PNPLA2 and HSL levels are closely associated with TG levels, adipocyte size, and metabolic parameters due to their role in regulating lipolysis^34–36^. However, in our study, microbiota depletion significantly decreased the blood TG level despite increasing PNPLA2 and HSL levels^37,38^. Based on these results, we hypothesized that the reduction in glucose availability in GF mice compensated for increased FFA oxidation for energy production via the upregulation of adipose tissue lipolysis. In addition, qPCR analysis showed that the expression of the *Pnpla2* gene and fatty acid oxidation marker genes was significantly increased in the gWAT of GF mice (Supplementary Fig. 10b, c). These observations suggest that increased fatty acid oxidation metabolism in GF mice is necessary to satisfy the metabolic demands caused by defective glucose metabolism.

## Discussion

Based on previous reports and our preliminary phenotype analysis, we observed that GF mice had a low body weight, low fat mass, and metabolically healthy blood indices in addition to a low oxygen consumption rate and low ATP content in skeletal muscle (Fig. 1). In particular, the low blood glucose levels in GF mice were well reflected in their increased blood glucagon concentrations. High levels of blood glucagon stimulate gluconeogenesis and glycogenolysis to increase blood glucose levels^39^. However, since aberrant blood glucose and glucagon levels were observed in GF mice under nonfasting conditions, we predicted that the energy metabolism of GF mice would differ from that of SPF mice. GF mice are resistant to diet-induced obesity, with specifically impaired lipid digestion and absorption. Therefore, high-fat diet-treated GF mice displayed reduced levels of fasting blood glucose and insulin compared with SPF mice^40^, similar to our results. These previous results and our results suggest that GF mice are in an energy-deficit condition, as evidenced by low blood glucose, low insulin, and high glucagon levels. We aligned our research to investigate this hypothesis since GF mice exhibited lower oxygen consumption despite their metabolically healthy phenotype. We were able to explore the process of energy metabolism in GF mice by altering oxygen consumption and energy metabolism through an exercise challenge.

Exercise capacity may be directly controlled by the gut microbiota, as it is a major factor in energy production via food digestion and absorption^41–43^. Recent studies have demonstrated that the gut microbiota controls glucose homeostasis by modulating bile acid metabolism, the gluconeogenic pathway, and selective glucose uptake into brown and white adipose tissues^20,22,32,33^. However, no investigations into energy consumption under microbial depletion settings have been performed; thus, information on physiological and molecular mechanisms involved in the control of the microbiota that affect exercise ability remains limited. It was previously reported that when GF mice were subjected to a single bout of swimming exercise, the duration of exercise was significantly shorter in GF mice than in SPF mice and a *Bacteroides fragilis* gnotobiotic model^17^. The authors also reported that, despite six weeks of swimming exercise training, GF mice did not display enhanced exercise performance and that GF mice had low blood glucose levels and low GLUT4 expression in skeletal muscle^18^. However, the physiological and molecular mechanisms have not been elucidated to systematically understand the metabolic phenotypes of GF mice. Our study is the first to show that defective glucose metabolism is linked to impaired exercise capacity in GF mice. We performed aerobic exercise training in both SPF and GF mice to better understand how microbiota depletion affects host energy availability. Additionally, we observed how GF mice reacted to exercise training and how their energy production differed from that of SPF mice.

Interestingly, GF mice ran less than half of the distance run by SPF mice, and despite eating much more food, there was no change in the weight gain ratio or fat mass between the two groups. Our earlier investigation found that during voluntary wheel running, SPF mice consumed considerably more food than control mice^44,45^. It is clear that this higher food intake generates the energy required for continuous activity. Monitoring the caloric content of feces showed that GF mice excreted more calories than SPF mice, which could explain why they consumed more food despite running a shorter distance. The lower training effort of GF mice resulted in lower oxygen uptake capacity than of SPF mice. We were very careful in interpreting these results because SPF and GF mice differ in their oxygen consumption at basal levels. We performed an exhaustion test after 4 weeks of training to achieve a more definitive physiological conclusion on the training impact and confirmed once more that exercise training did not influence the exercise performance of GF mice. These data revealed that the microbiota plays an essential role in the exercise-induced increase in aerobic endurance performance.

Despite the decreased exercise capacity and oxygen uptake ability of GF mice, the blood lactate concentration was not elevated even after fatiguing exercise, which is a significant finding of our study. Increased blood lactate levels are known to be negatively correlated with VO_2max_ and endurance running performance^46^. As a result, lactate clearance in skeletal muscle is increased following endurance exercise, resulting in lower blood lactate levels in well-trained individuals. Thus, we inferred that insufficient glycogen stores in GF mouse skeletal muscle were the cause of their low blood lactate levels despite running to exhaustion.

Our interpretation is based on reports that well-trained athletes have better athletic performance due to increased glycogenolysis and glycolysis^47^ and that exercise-induced blood lactate levels are reduced in mice lacking liver glycogen synthase (LGS)^48^. Glycolysis is important for muscle power generation, and lactate is also a source of fuel energy^27,47,49^. Based on strong evidence from animal and human studies that glycolysis and glycogen catabolism proceed with lactate production under fully aerobic conditions^27,50^, the impaired exercise performance in GF mice was interpreted as a combination of low glycolytic and low oxygen consumption capacities. Furthermore, despite having considerably greater systemic glucose tolerance and insulin sensitivity than SPF mice, which had a high level of Akt activation in iWAT, GF mice had a low level of Akt phosphorylation in skeletal muscle. These findings explain why the ability of GF mice to absorb glucose into the muscle was disrupted, resulting in decreased glycolysis and glycogen storage. Interestingly, we found that GF mice cohoused with SPF mice exhibited increased exercise training volume due to the recovered glucose metabolism, which led us to the conclusion that the whole-body aerobic exercise capacity level was restored to the level of that in SPF mice. We reported the results of experiments in which GF mice were cohoused with SPF mice for four weeks to investigate the reversibility of glucose metabolism and exercise capacity. As a result, the previously documented phenomenon of reduced exercise ability in GF mice was completely reversed after 4 weeks of cohousing with SPF mice.

We also hypothesized that GF mice would adapt to metabolic demands by increasing fat utilization to compensate for impaired glucose metabolism. This hypothesis was supported by previous data suggesting that insufficient glucose metabolism would be supplemented by the activation of fat metabolism. Low adipose tissue size and very low TG levels in the liver and skeletal muscle were also observed in GF mice. Furthermore, prior studies have shown that GF mice are leaner than SPF mice with normal microbiota and accumulate less fat when subjected to diet-induced obesity^19–21^. Similarly, the levels of adipose-browning marker genes were robustly enriched in the iWAT of GF mice, as were the levels of genes in lipid oxidation- and fatty acid oxidation-related pathways.

Although our study is the first to show that decreased exercise capacity in GF mice is caused by a loss in glycogen storage capacity in skeletal muscle, we did not conduct experiments to identify specific bacteria that could affect this phenotype. Therefore, future studies on specific microbiota that can increase exercise capacity by modulating glucose metabolism should be performed.

**Fig. 7:**
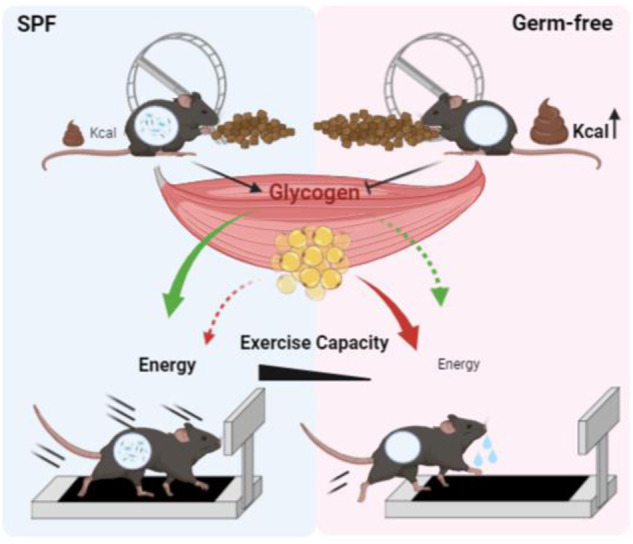
The microbiota is an essential regulator of exercise capacity. Skeletal muscle glycogen is the main substrate that determines exercise capacity. In this study, we revealed that the differences in exercise capacity between SPF and GF mice were related to glucose metabolism and glycogen storage capacity in skeletal muscle. Trained SPF and GF mice ate more food than untrained mice, but GF mice exhibited lower calorie absorption than SPF mice because their feces contained significantly more calories. GF mice exhibited a diminished exercise capacity and ability to adapt to voluntary wheel running exercise training relative to SPF mice. Decreased skeletal muscle glucose metabolism and glycogen storage were associated with impaired exercise capacity in GF conditions. GF mice consumed more energy through increased usage of fat as an alternative energy source and increased adipose browning ability, which is thought to impair ATP production, causing decreased exercise capacity.

In conclusion, our study underlines the significance of the microbiota in modulating exercise capacity via glucose metabolism. Using GF mice, we showed that microbiota depletion impairs glucose absorption and storage capacity in skeletal muscle, reducing exercise capacity. Furthermore, GF mice used more energy due to increased fat usage as an alternate energy source and enhanced adipose browning ability, which is likely to hamper ATP synthesis, resulting in decreased exercise capacity (Fig. 7).

## Supplementary information

Supplementary Figures

## Data Availability

Data supporting the study findings are available from the corresponding authors upon reasonable request.
