# Supplementary material for: Microbiota influences host exercise capacity via modulation of skeletal muscle glucose metabolism in mice
Source: Exp Mol Med. 2023 Aug 4;55(8):1820–30. doi: 10.1038/s12276-023-01063-4 (PMC10474268; doi:10.1038/s12276-023-01063-4)
Supplement: Supplementary file 1 — Supplementary Figures [file 12276_2023_1063_MOESM1_ESM.pdf]

Supplementary Figure

Microbiota influences host exercise capacity via modulation of skeletal muscle glucose metabolism in mice

Kim et al.,

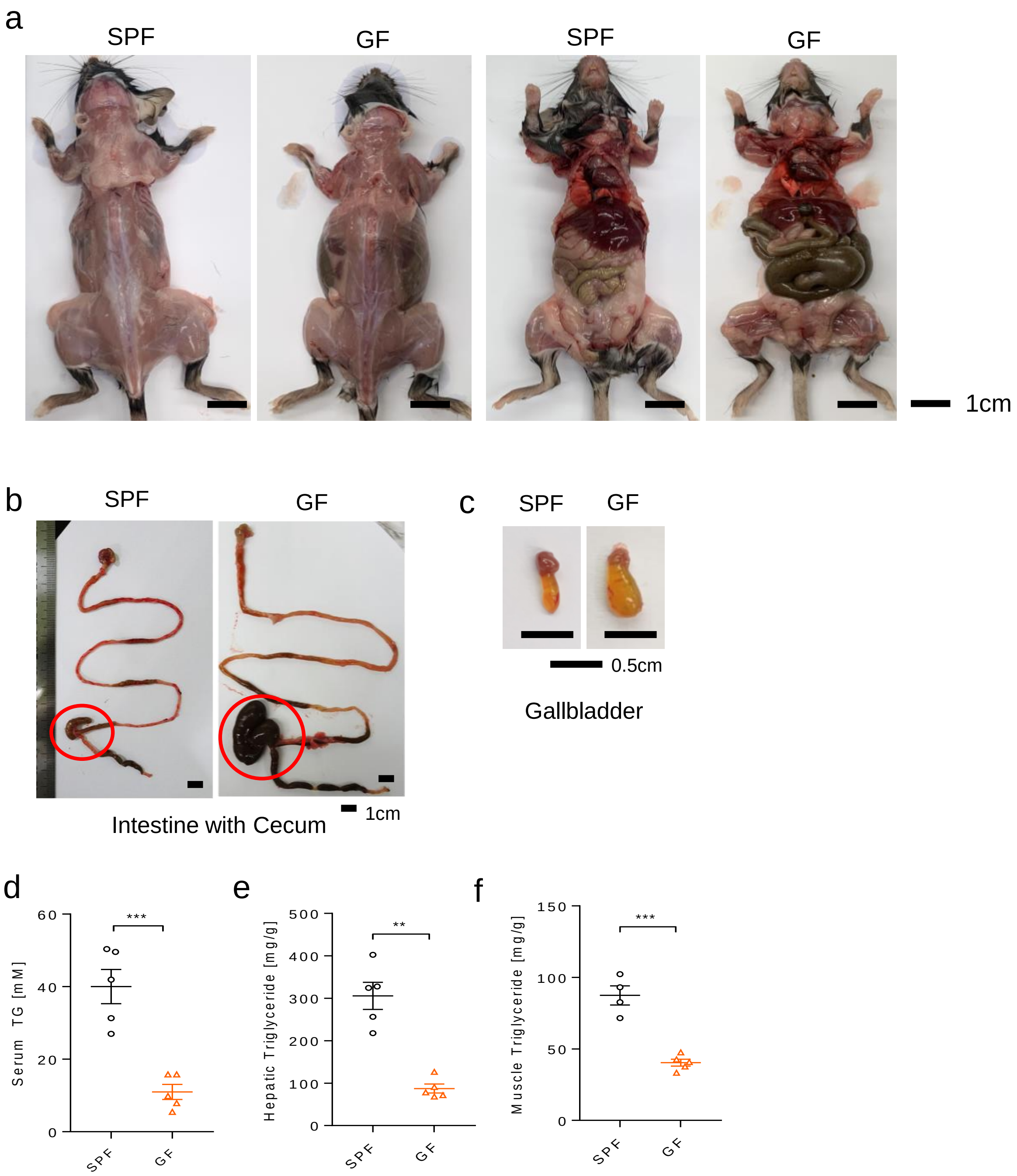

Supplementary Fig. 1 Phenotype of Germ-free mice at 12 weeks-old. (a) Body gross of SPF and GF mice. (b) Representative image of intestine and cecum in SPF and GF mice. (c) Gall bladder of SPF and GF mice. (d) Serum triglyceride (TG) level [mM]; n = 5 for all groups. (e) Hepatic triglyceride [mg/g]; n = 5 for all groups. (f) Muscle triglyceride [mg/g]; SPF, n = 4; GF, n = 5.

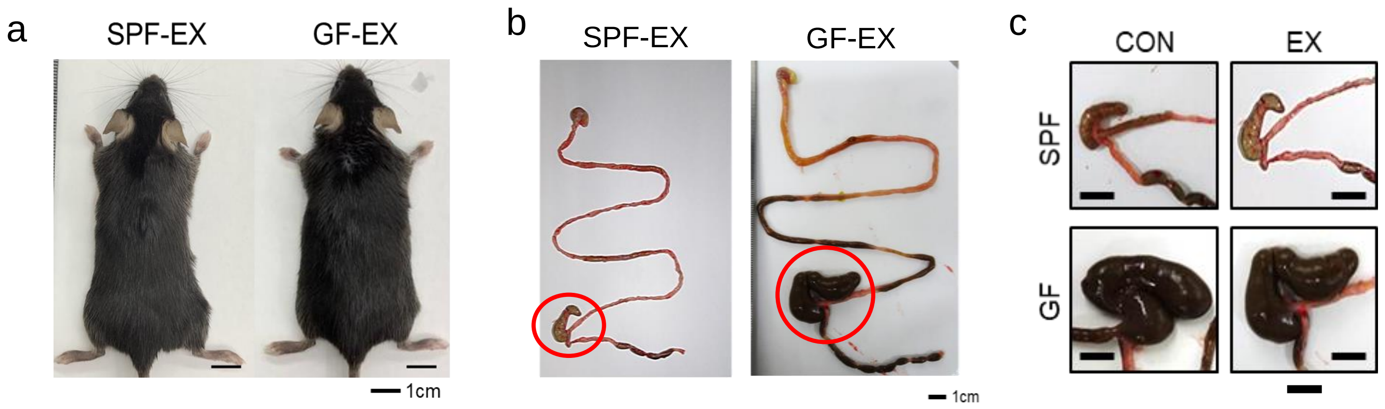

Supplementary Fig. 2 Germ-free mice have large cecum and gallbladder. (a) Body gross of exercised SPF and GF mice. (b) Representative image of intestine exercised SPF and GF mice.(c) Cecum of exercised SPF and GF mice. (a-f) n=3 for all group (a-e, scale bar=1cm; f, scale bar=0.5cm). (a-b) n=3 for all group (a-e, scale bar=1cm; f, scale bar=0.5cm).

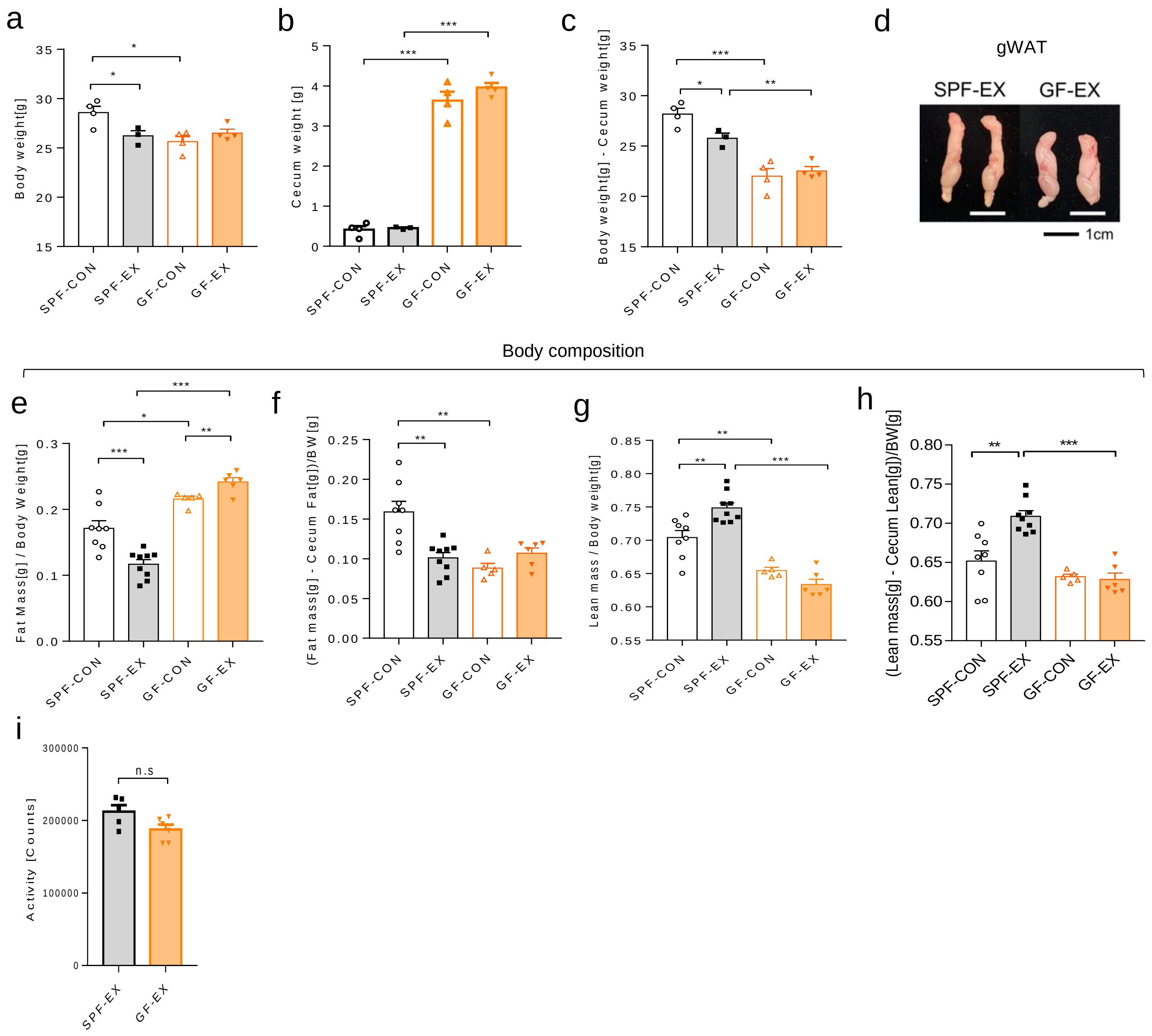

Supplementary Fig. 3 Metabolic Phenotypes of exercise trained SPF and GF mice. (a) Body weight [g] (b) Cecum weight [g] (c) Body weight [g] – cecum weight [g] (a-c) SPF-CON, n=4; SPF-EX, n=3; GF-CON, n=4; GF-EX, n=4. (d) Representative image of gWAT in exercised SPF and GF mice; n=3 for all group. (e) Fat mass [g] / body weight [g] (f) (Fat mass [g] – cecum mass [g]) / body weight [g] (g) Lean mass [g] / body weight [g] (h) (Lean mass [g] – cecum mass [g]) / body weight [g] (e-h) SPF-CON, n=8; SPF-EX, n=9; GF-CON, n=5; GF-EX, n=6. (i) Activity [counts] after exercise; SPF-EX, n = 5; GF-EX, n = 6. Values are expressed as mean ± SEM. Differences between two groups were analyzed using two-tailed Student’s t-test. Differences between the groups were analyzed using one-way ANOVA, post-hoc Tukey’s test. *p<0.05, **p<0.01, ***p< 0.001.

a

	min (cumulative)	cm/s	m/min
Calibration	30	0	0
Adaptation	5	5.00	3
		8.33	5
		11.67	7
		15.00	9
TEST	3 (3)	16.67	10
	3 (6)	23.33	14
	3 (9)	30.00	18
	3 (12)	36.67	22
	3 (15)	43.33	26
	3 (18)	50.00	30
	3 (21)	56.67	34
	3 (24)	63.33	38
	3 (27)	70.00	42
	3 (30)	76.67	46
Recovery	20	0	0

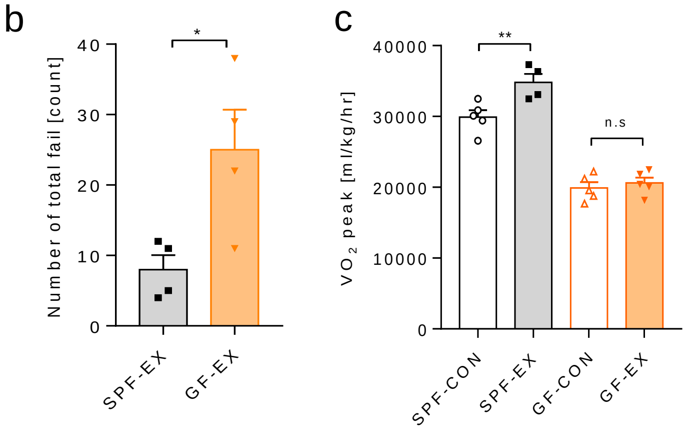

Supplementary Fig. 4 GF mice display impairment exercise capacity (a) A treadmill exhaustion test protocol (b) Total fail counts during exhaustion test in trained SPF and GF mice; n=4 for all group. (c) VO_2 peak [mL/kg/h] during exhaustion test.; SPF-CON, n=5; SPF-EX, n=4; GF-CON, n=5 GF-EX, n=5. Values are expressed as mean ± SEM. Differences between two groups were analyzed using two-tailed Student's t-test. Differences between the groups were analyzed using one-way ANOVA, post-hoc Tukey's test. *p<0.05, **p<0.01, ***p< 0.001.

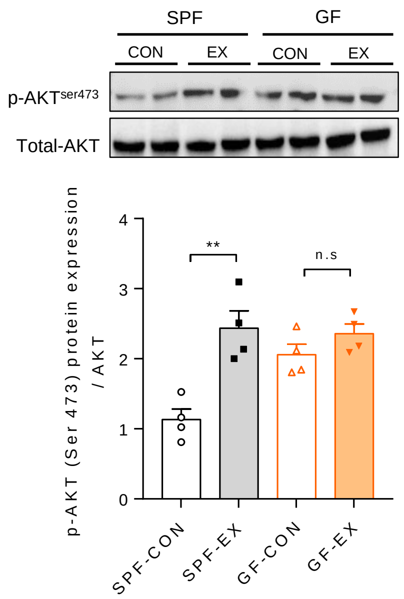

Supplementary Fig. 5 GF mice promote AKT phosphorylation in iWAT. p-AKT (Ser473) levels of iWAT in exercised SPF and GF mice after insulin (1U/kg) intraperitoneal (ip) injection; n = 4 for all groups. Protein expression was quantified based on AKT protein level. Values are expressed as mean ± SEM. Differences between two groups were analyzed using two-tailed Student's t-test. Differences between the groups were analyzed using one-way ANOVA, post-hoc Tukey's test. *p<0.05, **p<0.01, ***p< 0.001.

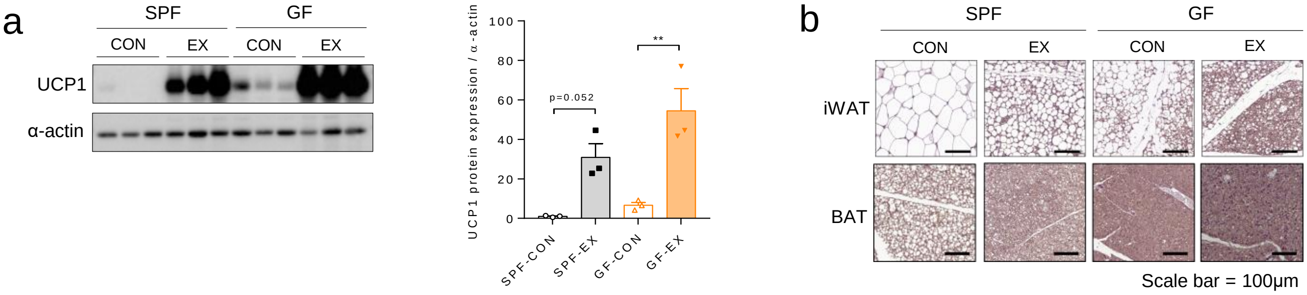

Supplementary Fig. 6 Increased adipose tissue browning in GF mice. (a) UCP1 protein expression of iWAT after exercise in SPF and GF mice. UCP1 protein expression were quantified by α-actin protein level; n = 3 for all groups. (b) UCP1 IHC staining of iWAT and BAT after exercise in SPF and GF mice. n=3 for all groups; scale bar = 100 μm.

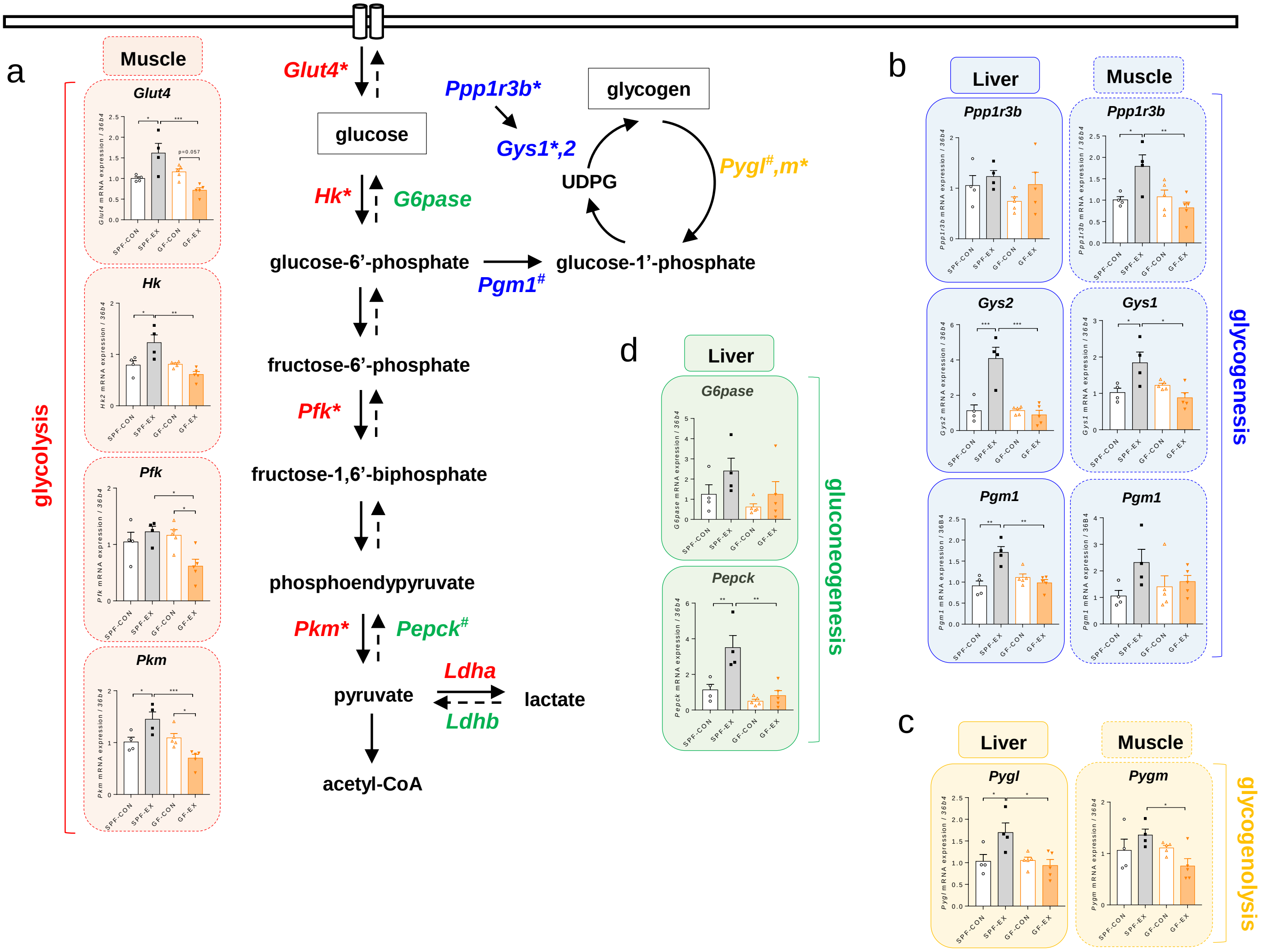

Supplementary Fig. 7 Microbiota depletion impairs glucose metabolism in skeletal muscle (a) Glycolysis related gene expression levels in gastrocnemius muscle after exercise in SPF and GF mice. (b) Glycogenesis related gene expression levels in liver and gastrocnemius muscle after exercise in SPF and GF mice. (c) Glycogenolysis related gene expression levels in liver and gastrocnemius muscle after exercise in SPF and GF mice. (d) Gluconeogenesis related gene expression levels in liver after exercise in SPF and GF mice. (a–d), SPF-CON, n = 4; SPF-EX, n = 4; GF-CON, n = 5; GF-EX, n = 5. (a-d) The expression of the target genes was normalized to that of 36B4. Values are expressed as mean ± SEM. Differences between two groups were analyzed using two-tailed Student's *t*-test. Differences between the groups were analyzed using one-way ANOVA, post-hoc Tukey's test. *p<0.05, **p<0.01, ***p< 0.001.

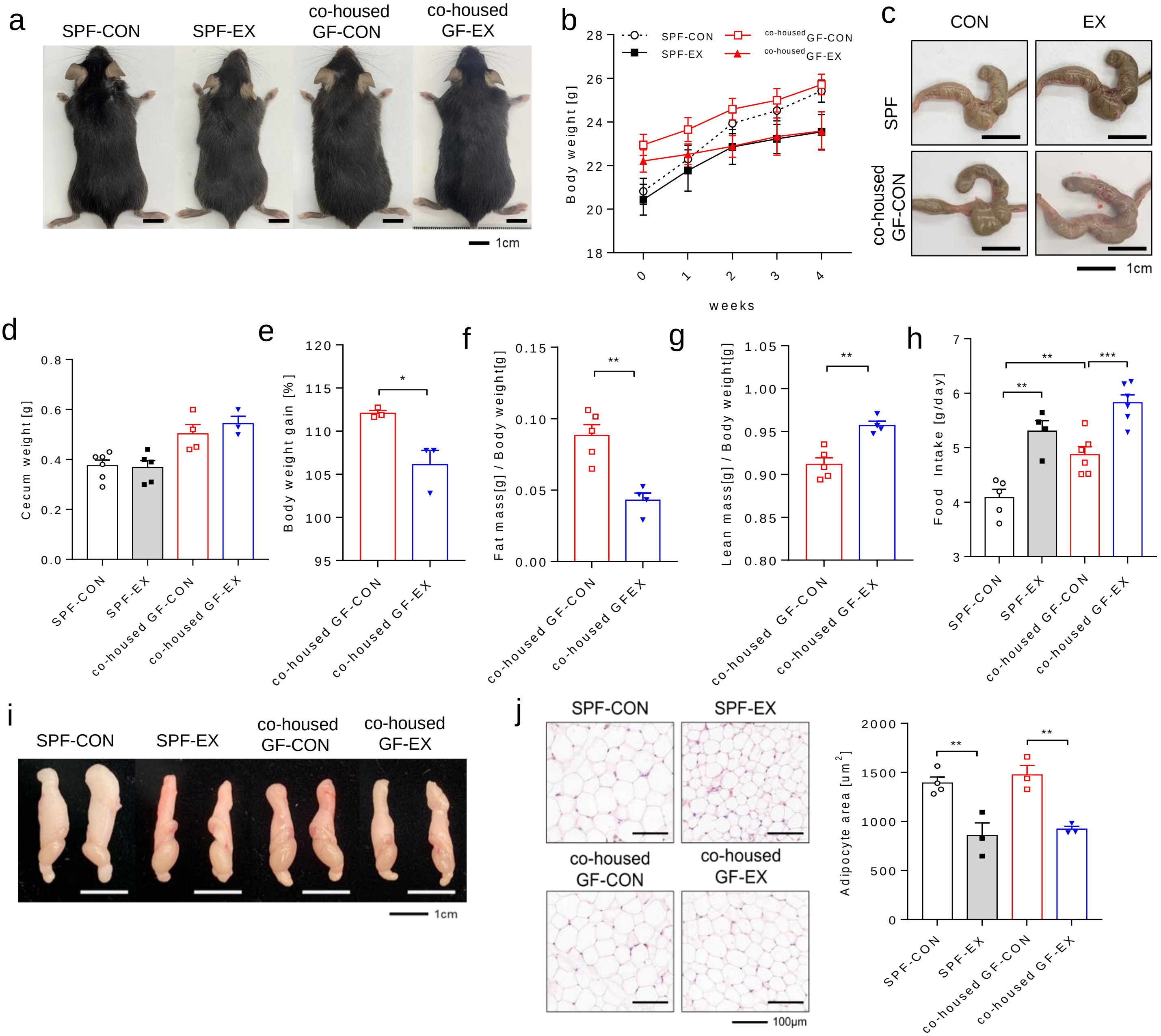

Supplementary Fig. 8 Phenotype of GF $^{\text{co-housed}}$ mice. (a) Body gross of exercised SPF and GF $^{\text{co-housed}}$ mice. (b) Body weight [g] during exercise; SPF-CON, n=6; SPF-EX, n=6; GF-CON $^{\text{co-housed}}$, n=3; GF-EX $^{\text{co-housed}}$, n=3 (c) Cecum of exercised SPF and GF co-housed mice. (a and b) n=3 for all group (scale bar=1cm). (d) Body weight [g]-Cecum weight [g] after exercise; SPF-CON, n=6; SPF-EX, n=6; GF-CON $^{\text{co-housed}}$, n=3; GF-EX $^{\text{co-housed}}$, n=3 (e) Body weight gain [%]; n=3 for all group. (f) Fat mass [g] / body weight [g] using body composition. (g) Lean mass [g] / body weight [g] using body composition. (f and g) GF-CON $^{\text{co-housed}}$, n=5; GF-EX $^{\text{co-housed}}$, n=4. (h) Daily food intake [g/day]; SPF-CON, n=5; SPF-EX, n=4; GF-CON $^{\text{co-housed}}$, n=6; GF-EX $^{\text{co-housed}}$, n=6. (i) gWAT of exercised SPF and GF $^{\text{co-housed}}$ mice; n=3 for all group. (scale bar=1cm) (j) H&E staining of gWAT; n=3 for all group. (scale bar=100μm) and Adipocyte size distribution [μm^2]; SPF-CON, n=4; SPF-EX, n=3; GF-CON $^{\text{co-housed}}$, n=3; GF-EX $^{\text{co-housed}}$, n=4. Values are expressed as mean ± SEM. Differences between two groups were analyzed using two-tailed Student's t-test. Differences between the groups were analyzed using one-way ANOVA, post-hoc Tukey's test. *p<0.05, **p<0.01, ***p< 0.001.

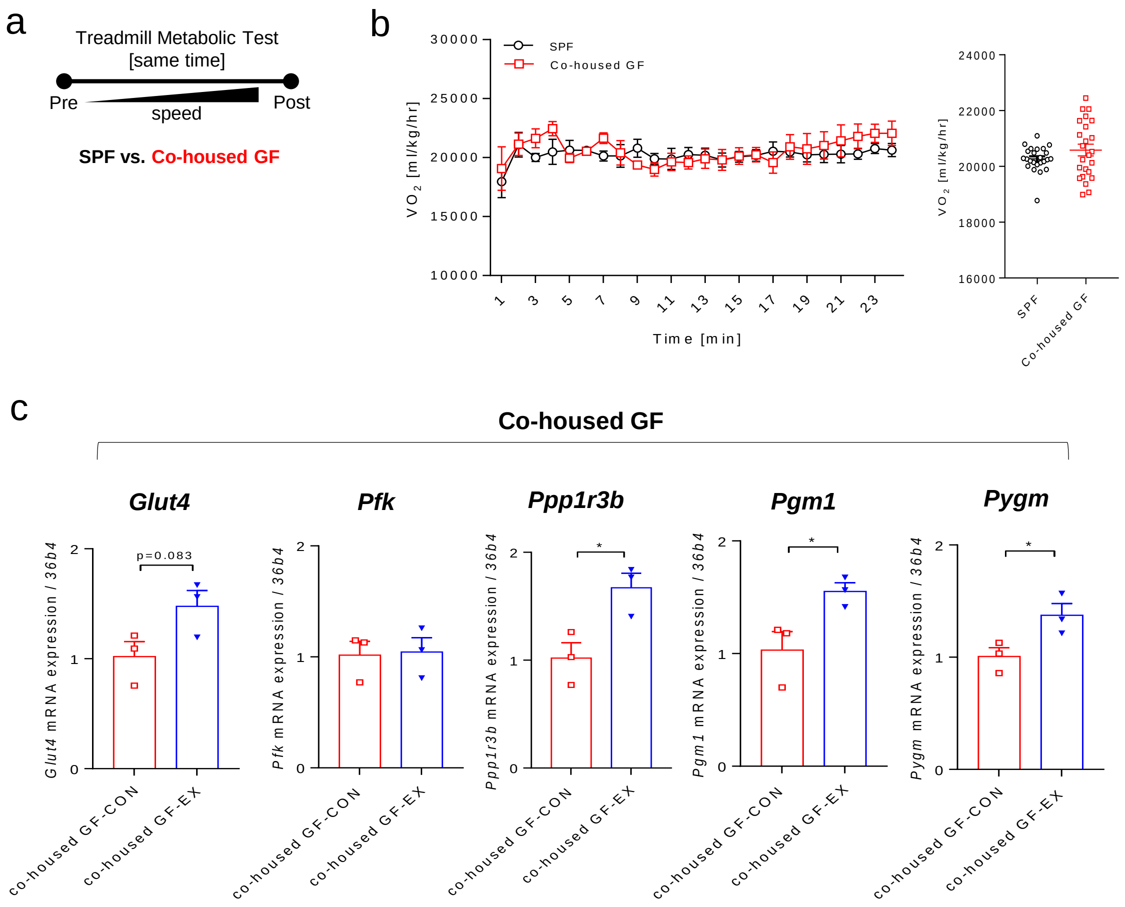

Supplementary Fig. 9 GF co-housed mice display rescued exercise capacity and glucose metabolism. (a) Experimental design of treadmill exhaustion metabolic test in SPF and co-housed GF mice. (b) VO_2 [mL/kg/h] during exhaustion test; SPF, n=5; co-housed GF, n=4. (c) Glycolysis, glycogenesis and glycogenolysis related gene expression levels in gastrocnemius muscle after exercise in SPF and co-housed mice; n = 3 for all groups. Values are expressed as mean ± SEM. Differences between two groups were analyzed using two-tailed Student's t-test. Differences between the groups were analyzed using one-way ANOVA, post-hoc Tukey's test. *$p<0.05$, **$p<0.01$, ***$p<0.001$.

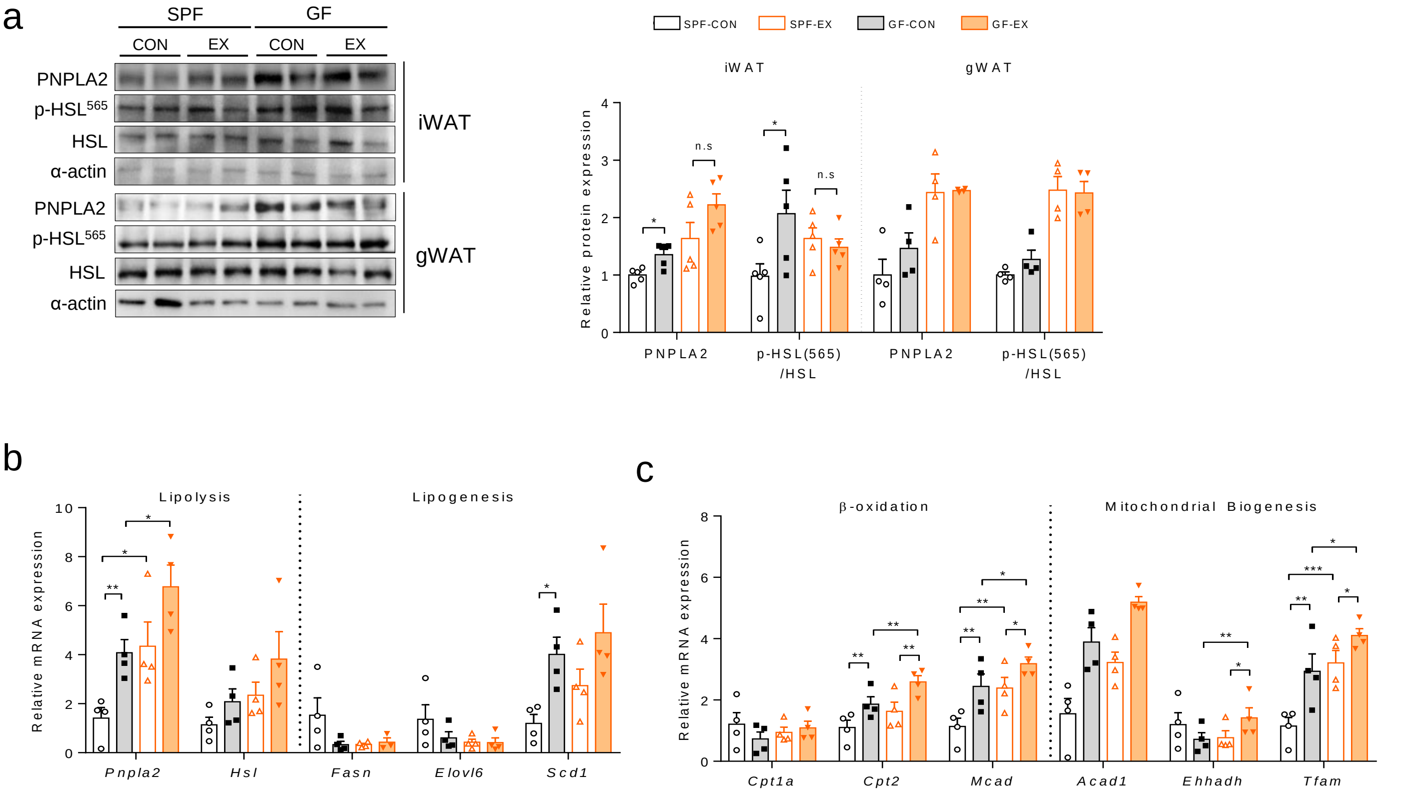

Supplementary Fig. 10 Increased adipose tissue oxidative capacity in GF mice. (a) Lipolysis related protein expression of iWAT and gWAT after exercise in SPF and GF mice. PNPLA2 protein expression was quantified based on α-actin protein level and p-HSL (Ser565) protein expression was quantified based on total HSL protein level; n = 5 for all group. (b) Lipolysis and lipogenesis related gene expression of gWAT in exercised SPF and GF mice; n=4 for all group. (c) *β*-oxidation and mitochondrial biogenesis related gene expression of gWAT in exercised SPF and GF mice; n=4 for all group. Values are expressed as mean ± SEM. Differences between two groups were analyzed using two-tailed Student's t-test. Differences between the groups were analyzed using one-way ANOVA, post-hoc Tukey's test. **p*<0.05, ***p*<0.01, ****p*< 0.001.

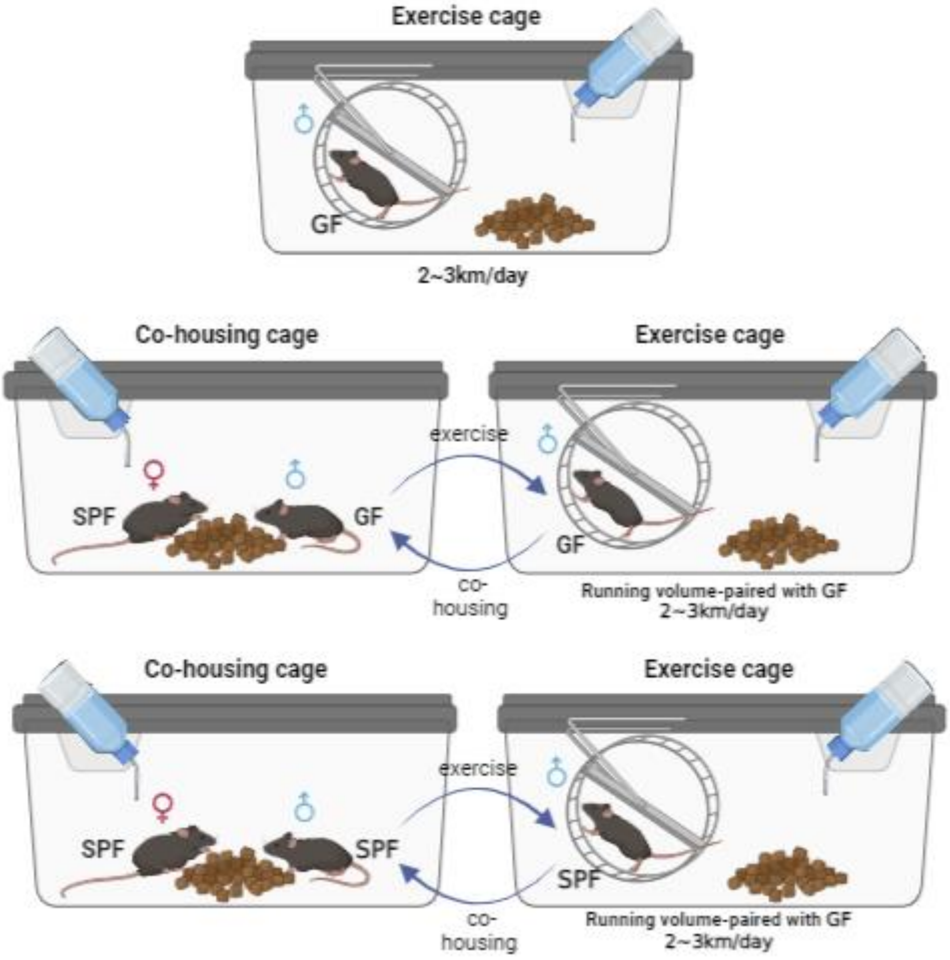

Supplementary Fig. 11 A schematic illustration of the paired SPF or paired co-housing GF voluntary wheel running exercise training

	Forward (5'→3')	Reverse (5'→3')
Acad1	CTTGGGAAGAGCAAGCGTACT (21mer)	CTGTTCTTTTGTGCCGTAATTCG (23mer)
Pnpla2	CATGATGGTGCCCTATACTCTG (22mer)	CTACCCGTCTGCTCTTTCATC (21mer)
Cd36	TGGCCTTACTTGGGATTGG (19mer)	CCAGTGTATATGTAGGGCATCCA (24 mer)
Cpt1a	TCGATCTCCGCCTGAGCCATGA (22mer)	TCGCGGGGAACACACCAGTGAT (22mer)
Cpt2	GCTCCGAGGCATTTGTC (17mer)	CATCGCTGCTTCTTTGGT (18mer)
Ehhadh	CCAATGCAAAGGCTCGTGTT (20mer)	GGTAGAAGCTGCGTTCCTCTTG (22mer)
Elovl6	GAAAAGCAGTTCAACGAGAACG (22mer)	AGATGCCGACCACCAAAGATA (21mer)
Fasn	GGAGGTGGTGATAGCCGGTAT (21mer)	TGGGTAATCCATAGAGCCCAG (21mer)
G6pase	GTCGACTCGCTATCTCCAAG (20mer)	GCAATGCCTGACAAGACTCC (20mer)
Glut2	GCACAGACACCCCACTTACA (20mer)	GCAAAGCTGGACACAGACA (19mer)
Glut4	GGTGTGGTCAATACGGTCTTCAC (23mer)	AGCAGAGCCACGGTCATCAAGA (22mer)
Gys1	GAGAACGCAGTGCTTTTCGA (20 mer)	TCATCCCCTGTCACCTTCG (19mer)
Gys2	CCAGCTTGACAAGTTCGACA (20 mer)	ATCAGGCTTCCTCTTCAGCA (20 mer)
Hk	TCCAGACGGTACAGAGAAAGGA (22mer)	TCTCTACGCCCCTTCGCTTG (20mer)
Hsl	ATGCCACTCACCTCTGATCC (20mer)	CTGTCCTGTCCTTCCCGTAG (20mer)
Mcad	ACTGCCCGCAAGTTTGCCAGA (21mer)	TGTGCGCGTTGATCAAGCCGA (21mer)
Pepck	CACCTCCTGGAAGAACAAGG (20mer)	CTACGGCCACCAAAGATGAT (20mer)
Pfk	GGAGTGCGTGCAGGTGACCAAA (22mer)	ATCACGGCCACTGTGTGCAACC (22mer)
Pgm1	AGCCAATGACCCAGATGCTGAC (22mer)	TCCAGGAAGTGAAGAGCCACCA (22mer)
Pkm	GGCTCACACAGGTTCTCTTT (20mer)	TCTCGTCTTGACCACATCAAC (21mer)
Ppp1r3b	GAATGCGGGTTTAAGCATCTATC (23mer)	CCGCTTCTTCACCTTCTTCT (20mer)
Pygl	GGCAGAAGTGGTGAACAATGACC (23mer)	TCCGATAGGTCTGTGGCTGGAA (22mer)
Pygm	ATGGCACACCTGTGCATTGCTG (22mer)	CGAGGAGTGATGCCATTGGTCT (22mer)
Scd1	TTCTTGCGATACACTCTGGTCG (22mer)	CGGGATTGAATGTTCTTGTCGT (22mer)
Tfam	AAGACCTCGTTCAGCATATAACATT (25mer)	TTTTCCAAGCCTCATTTACAAGC (23mer)
36b4	GAGGAATCAGATGAGGATATGGGA (24mer)	AAGCAGGCTGACTTGGTTGC (20mer)

Supplementary Table. 1 Primer sequence.
